# Mechanisms Regulating Skin Pigmentation: The Rise and Fall of Complexion Coloration

**DOI:** 10.3390/ijms10094066

**Published:** 2009-09-15

**Authors:** Jody P. Ebanks, R. Randall Wickett, Raymond E. Boissy

**Affiliations:** 1 Department of Pharmaceutical Sciences, University of Cincinnati College of Pharmacy, 136 Health Professions Building, 3225 Eden Ave., Cincinnati, OH 45267-0004, USA; E-Mails:ebanksjp@email.uc.edu (J.E.);wicketrr@ucmail.uc.edu (R.W.); 2 Department of Dermatology, University of Cincinnati College of Medicine, 231 Albert Sabin Way, ML-0592, Cincinnati, OH 45267-0592, USA

**Keywords:** depigmenting agents, hyperpigmentation, melanin, pigment, tyrosinase

## Abstract

Skin pigmentary abnormalities are seen as aesthetically unfavorable and have led to the development of cosmetic and therapeutic treatment modalities of varying efficacy. Hence, several putative depigmenting agents aimed at modulating skin pigmentation are currently being researched or sold in commercially available products. In this review we will discuss the regulation of processes that control skin complexion coloration. This includes direct inhibition of tyrosinase and related melanogenic enzymes, regulation of melanocyte homeostasis, alteration of constitutive and facultative pigmentation and down-regulation of melanosome transfer to the keratinocytes. These various processes, in the complex mechanism of skin pigmentation, can be regulated individually or concomitantly to alter complexion coloration and thus ameliorate skin complexion diseases.

## 1. Introduction

The acquisition of an aesthetically pleasing skin pigmentary appearance has been a primary focus of many cosmetic and therapeutic based industries. As a result, several treatment modalities are being investigated for their efficacy in treating skin hyperpigmentary lesions. This review will detail many of the current skin depigmenting agents and treatment approaches that are currently being employed to combat skin pigmentary disorders.

Melanocytes, the pigment producing cells of the follicular and interfollicular epidermis, produce a specialized lysosomal related organelle termed the melanosome. Within the melanosome, biopolymers of the pigment melanin are synthesized to give hair and skin, as well as other tissue, its color. This melanin synthesis involves a bipartite process in which structural proteins are exported from the endoplasmic reticulum and fuse with melanosome-specific regulatory glycoproteins released in coated vesicles from the Golgi-apparatus. Melanin synthesis ensues subsequent to the sorting and trafficking of these proteins to the melanosome [1,2]. Each melanocyte resides in the basal epithelial layer and, by virtue of its dendrites, interacts with approximately 36 keratinocytes to transfer melanosomes and protect the skin from photo-induced carcinogenesis. Furthermore, the amount and type of melanin produced and transferred to the keratinocytes with subsequent incorporation, aggregation and degradation influences skin complexion coloration [3]. Hyperpigmentary disorders of the skin such as melasma, agespots or solar lentigo can result from the overproduction and accumulation of melanin [4,5]. As such, depigmenting agents can have potent effects by acting on one or more steps in the melanogenic pathway, melanosome transfer or post-transfer pigment processing and degradation.

**Scheme 1.**
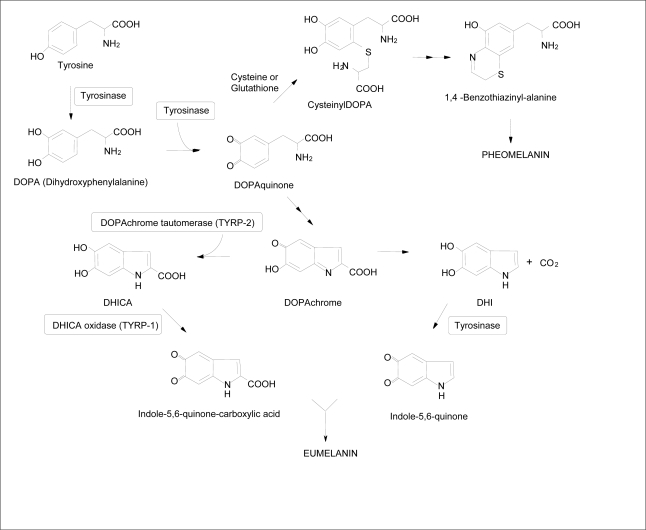
Process of melanogenesis within epidermal melanosomes. Tyrosinase, the rate limiting enzyme of melanogenesis, catalyzes the hydroxylation of l-tyrosine to DOPA and the oxidation of DOPA to DOPAquinone. If cysteine or glutathione is present, it reacts with DOPAquinone to produce cysteinylDOPA and the benzothiazine derivatives of pheomelanin. As cysteine is diminished, DOPAquinone cyclizes into DOPAchrome. TYRP-2 catalyzes the tautomerization of DOPAchrome to 5,6-dihydroxyindole-2-carboxylic acid (DHICA), which is later oxidized to DHICA-melanin subunits. The oxidation of DHICA to eumelanin is thought to be catalyzed by TYRP-1. In the absence of TYRP-2 the carboxylic acid moiety of DOPAchrome is spontaneously lost to form 5,6-dihydroxyindole (DHI). DHICA in conjunction with DHI comprise subunits of eumelanin [11,12].

Tyrosinase, the rate limiting enzyme of the melanogenic pathway, is a copper containing glycoprotein of approximately 60–70 kDa and a common target for therapeutic agents intended to alleviate cutaneous hyperpigmentation [6,7]. The biosynthesis of the two major forms of melanin, black/brown eumelanin and yellow/red pheomelanin is initially catalyzed by tyrosinase. Specifically, the enzyme catalyzes the hydroxylation of the monophenol l-tyrosine to the *o*-diphenol 3,4-dihydroxyphenylalanine (DOPA) and the oxidation of DOPA to the *o*-quinone DOPAquinone (Scheme 1) [8,9]. Several depigmenting agents modulate skin pigmentation by influencing the transcription and activity of tyrosinase as well as related melanogenic enzymes tyrosinase related protein-1 (TYRP-1), tyrosinase related protein-2 (TYRP-2) and/or peroxidase [10].

## 2. Transcriptional Regulation of Melanogenic Enzymes

The transcriptional level is the first stage by which the expression of tyrosinase and related melanogenic enzymes may be modulated. Influential in this process, the microphthalmia-associated transcription factor (MITF) is a basic helix-loop-helix leucine zipper transcription factor that regulates melanocyte cellular differentiation as well as the transcription of melanogenic enzymes (tyrosinase, TYRP1 and TYRP2) and melanosome structural proteins (MART-1 and PMEL17) [13–15]. Mutations in the MITF gene are associated with auditory and pigmentary abnormalities of Waardenburg syndrome type IIA [16,17].

The MITF-M isoform, with the promoter most proximally located upstream to the common exon sequences, is exclusively expressed in melanocytes and is believed to bind the M box regulatory element and transactivate the promoter of tyrosinase, TYRP-1 and TYRP-2, as well as other genes. [7,10,13,15,18,19]. It is believed that upon stimulatory binding of α-melanocyte stimulating hormone (α-MSH) to the melanocortin 1 receptor (MC1R), adenyl cyclase is activated and cAMP produced. cAMP then activates the protein kinase A (PKA) pathway to phosphorylate cAMP-responsive element binding protein (CREB) transcription factors, which mediates MITF-M promoter activation to induce melanogenesis. MITF is also regulated at the transcriptional level by interleukin-6 (IL-6) and Wnt Signaling pathway. Furthermore, MITF is post-transcriptionally regulated by phosphorylation via ribosomal S6 kinase (RSK), glycogen synthase kinase-3b (GSK3b), p38 stress signaling and mitogen-activated protein kinase (MAPK) pathways by currently undefined mechanisms/pathways [15,18,20,21]. As MITF is considered an important regulator of melanogenesis, manipulation of the aforementioned signaling pathways may have potential therapeutic use.

Transforming growth factor-β1 (TGF-β1) is a cytokine that plays a role in cell differentiation, proliferation and apoptosis, in addition to the inhibition of pigmentation. TGF-β1 is believed to mediate the down-regulation of the MITF promoter activity, reducing the production of tyrosinase, TYRP-1, TYRP-2 and MITF protein levels [20,22]. Yang *et al.* have shown that TGF-β1 inhibits the expression of paired-box homeotic gene (PAX 3), a transcription factor and key regulator of MITF in melanocytes [23]. Kim *et al.* have also demonstrated that TGF-β1 influences the extracellular-signal related kinase (ERK) pathway and down-regulates MITF as well as melanogenic enzyme production [20,22,24,25]. Similarly, ERK activation by sphingosine-1-phosphate, C_2_-ceramide and sphingosylphosphorylcholine has also been reported by Kim *et al.*, which the authors hypothesize, may play an important role in the inhibition of melanogenesis [25–27]. ERK activation, in those cases, is thought to result in phosphorylation of MITF and its subsequent ubiquitination and degradation [25,28–30]. In a distinct study, the authors demonstrated that lysophosphatidic acid (LPA), a serum phospholipid released by activated platelets, mediates the reduction of MITF promoter activity as well as MITF and tyrosinase protein and melanin synthesis in Mel-Ab cells, an immortalized mouse melanocyte line [31].

In addition to the process of melanization, MITF also regulates melanocyte differentiation, development, and survival [32]. Pertaining to survival, MITF regulates the anti-apoptotic molecule Bcl-2 as well as additional survival genes [33,34]. It has recently been demonstrated that melanocytes deficient in MITF expression are compromised in their resistance to UV-induced apoptosis [35]. Therefore, caution is warranted when attempting to decrease skin pigmentation by down-regulating MITF since melanocyte death may be a consequence.

## 3. Post-Translational Modification of Melanogenic Enzymes

A major post-translational modification of melanogenic enzymes is the attachment of *N*-linked glycans to asparagine residues in Asn-X-Ser/Thr motifs (where X is not Pro), during the polypeptides translocation in the ER. This glycosylation is critical for the proper maturation of tyrosinase [10,36,37]. A detailed review of the processes involved in the *N*-glycosylation of melanogenic enzymes has been published by Branza-Nichita *et al.* [37]. Inhibition of proper *N*-glycan processing of melanogenic enzymes can result in improper polypeptide folding and in turn inhibition of melanogenesis, as they facilitate association with lectin-chaperones. Treatment with various agents that inhibit *N*-glycosylation can result in the down-regulation of melanosomal enzyme activity and reduced melanosomal maturation [9,10,37,38].

Studies completed by Mishima and Imokawa using tunicamycin and glucosamine, specific inhibitors of lipid carrier-dependent glycosylation of protein, resulted in decreased pigmentation and ultrastructural as well as biochemical aberrations in melanogenic compartments of treated B16 melanoma cells. In addition, electron microscopic analysis showed melanosomes with internal structural irregularities and pigment loss [10,39]. In a more recent study, Terao *et al.* tested a novel compound, BMY-28565, that inhibited melanogenesis by depressing tyrosinase activity with no impact on tyrosinase mRNA levels in B16 melanoma cells. As other active derivatives of the compound cause an increase in protein glycosylation in B16 melanoma cells, the authors hypothesize that the test compound inhibited tyrosinase by modifying the sugar moieties of the enzyme [40,41]. In a distinct study by Choi *et al.*, treatment of HM3KO melanoma cells with deoxynojirimycin, a α-glucosidase inhibitor that disrupts early ER *N*-glycan processing, and deoxymannojirimycin, an inhibitor of α-1,2-mannosidase which are thought to be responsible for late glycan processing, showed inhibition of glycosylation, transportation of tyrosinase to the melanosome and melanin synthesis [42]. Other factors explored for their ability to modulate tyrosinase glycosylation include calcium d-pantetheine-*S*-sulfonate [43], ferritin [44] and glutathione [45]. Glutathione induced inhibition of tyrosinase glycosylation, blocks the maturation and transfer of tyrosinase from GERL (Golgi-endoplasmic reticulum-lysosome)-coated vesicles to the pre-melanosome. Yet, other mechanisms of action proposed for glutathione include (A) the direct inactivation of tyrosinase by chelating copper within the enzyme’s active site, (B) mediating the transition from eumelanogenesis to pheomelanogenesis, as glutathione participates in the conversion of dopaquinone to pheomelanin, (C) antioxidant properties that quench free radicals and peroxides that induce melanin formation and (D) modulating the depigmenting capabilities of melanocytotoxic agents [20,46].

## 4. Attenuation of Tyrosinase and Related Melanogenic Enzymes Catalytic Activity

### 4.1. Hydroquinone

Hydroquinone (1,4-dihydroxybenzene, HQ) has been the gold standard for treating hyperpigmentation for more than 50 years and has been successfully used to treat melanosis. The compound can be found in wheat, tea, berries, beer and coffee, but is detoxified within the liver into inert compounds [6,47]. Hydroquinone is a phenolic compound and depigmenting agent that mainly exerts its effect on melanocytes with active tyrosinase. As HQ dependent melanogenic inhibition requires the presence of active tyrosinase, it is therefore not useful in altering the color of melanin that is previously present in the dermis and epidermis [48].

The structural similarity between HQ and melanogenic precursors enables HQ’s interaction with tyrosinase. This interaction mediates HQ’s inhibition of tyrosinase by binding histidines or copper at the active site of the enzyme. Additionally, HQ induced generation of reactive oxygen species and quinones leads to the oxidative damage of membrane lipids and proteins such as tyrosinase. Hydroquinone is also thought to inhibit pigmentation by depleting glutathione, modifying melanosome formation or reducing DNA and RNA synthesis with concomitant melanosome degradation and melanocyte destruction [10,20,49,50].

Traditional hydroquinone formulations contain other constituents that promote a synergistic effect. A popular formulation is comprised of hydroquinone and a corticosteroid to reduce inflammation, along with tretinoin, shown to reduce the atrophy associated with the corticosteroid and remove pigmentation by increasing keratinocyte turnover and the penetration of hydroquinone [50,51].

Due to the risks of side effects such as exogenous ochronosis and permanent depigmentation following long term use, hydroquinone has been banned by the European Committee (24^th^ Dir 2000/6/EC) and formulations have been withdrawn from cosmetics and are available only through prescription [10,20,50].

Other phenolic compounds have been evaluated for their depigmenting capabilities. In fact the chemical structures of several phenolic compounds have been investigated to delineate structure related tyrosinase inhibitory activity. It has been suggested that having a hydroxyl group *para* to an electron donator group is required for a compound to be an effective alternative substrate for tyrosinase [10]. Distinct structure-activity based analysis done by Ni-Komatsu *et al.* on quinolines, which contain a 4-substituted amino group with a tertiary amine side chain, shows significant inhibitory effect. Yet these quinolines, such as chloroquine, were not reported to influence the enzymatic activity of tyrosinase, but rather the intracellular trafficking of tyrosinase related proteins and lysosome associated membrane protein-1 (LAMP-1) [52].

### 4.2. Monobenzylether

The mono benzyl ether of hydroquinone (MBEH) is a related compound that is metabolized within the cell to form a quinone species that interacts with and results in permanent depigmentation, even at areas distant from the site of application. MBEH can destroy melanocytes and should not be used to treat post-inflammatory hyperpigmentation or melasma. MBEH therapy is appropriate for generalized depigmentation in the treatment of patients with vitiligo unresponsive to repigmentation therapy [10,20,48]. Proposed mechanisms of action for MBEH are both cytotoxicity to melanocytes as a result of free radical formation and competitive inhibition of tyrosinase activity [6].

### 4.3. Arbutin and Deoxyarbutin

Arbutin (hydroquinone-*O*-β-d-glucopyranoside), a derivative of hydroquinone, is a botanically derived compound found in cranberries, blueberries, wheat and pears [6,51]. Arbutin is used as an effective treatment of hyperpigmentary disorders, and displays less melanocyte cytotoxicity than hydroquinone. The compound inhibits melanogenesis by competitively and reversibly binding tyrosinase without influencing the mRNA transcription of tyrosinase. It also inhibits the maturation of melanosomes, possibly by its reported influence on DHICA polymerase activity and Pmel-17 protein. The mild effect of arbutin is attributed to the controlled release of hydroquinone as a result of *in-vivo* cleavage of the glycosidic bond. Higher concentrations of arbutin are more efficacious than lower concentrations, but may cause paradoxical hyperpigmentation [20,45,49,51,53]. Deoxyarbutin (dA), a synthetic form of arbutin synthesized without the hydroxyl moiety, provides a promising treatment for reducing skin hyperpigmentation [50]. dA shows reversible inhibition of tyrosinase activity with associated skin lightening in both a hairless guinea pig model system and in human skin. The reversibility of dA’s impact on skin pigmentation suggests that the compound does not permanently destroy melanocytes [20,54,55]. In addition to the reported efficacy, Hamed *et al.* have found that dA is less cytotoxic/cytostatic than HQ in treatment of cultured human melanocytes [56]. Chawla *et al.* have reported that dA and associated second-generation derivatives, dose-dependently inhibit tyrosinase hydroxylation and DOPAoxidase activity of tyrosinase. This may be attributed to the chemical structure of dA, as the deoxysugars may increase skin penetration and binding affinity for tyrosinase [20,54].

### 4.4. Mequinol

Mequinol (hydroquinone monomethyl ether, 4-hydroxyanisole, *para*-hydroxymethoxybenzene), another derivative of hydroquinone, is enzymatically oxidized by tyrosinase to produce melanocytotoxic quinones. The formation of quinones results in pigment cell destruction and skin depigmentation [10,57]. The combination of 0.01% tretinoin with mequinol has been reported to inhibit melanin production and has been shown to be effective and safe in the treatment of solar lentigenes and related hyperpigmentation [48,50,58,59].

### 4.5. N-Acetyl-4-S-Cysteaminylphenol

*N*-Acetyl-4-*S*-cysteaminylphenol (NCAP) is a phenolic thioether that has been used in the treatment of epidermal hyperpigmentation such as melasma and also in anti-melanoma studies. NCAP is structurally similar to tyrosine and acts specifically in melanin-synthesizing cells as an alternative tyrosinase substrate [48,50,60]. Ferguson *et al.* suggests that NCAP may undergo oxidation by tyrosinase to form a reactive *o*-quinone that is capable of alkylating thiol groups of essential enzymes, which may interfere with cell growth and proliferation [61]. In a 12 patient study of the efficacy of NCAP completed by Jimbow, the author describes an 8% complete loss and 66% marked improvement in visible changes of melanoderma after 2 to 4 weeks of topical application. The author attributes this depigmentation to a decrease in the number of functional melanocytes and the number of melanosomes transferred to keratinocytes. NCAP is suggested to be a depigmenting agent that is less irritating and more stable than HQ, with specificity for melanin-synthesizing cells [60].

### 4.6. Kojic Acid

Kojic acid (5-hydroxy-2-hydroxymethyl-4*H*-pyran-4-one, KA) is a naturally occurring hydrophilic fungal metabolite obtained from species of *Acetobacter, Aspergillus and Penicillium* [48,49]. Kojic acid is believed to inactivate tyrosinase by chelating copper atoms as well as suppressing the tautomerization of dopachrome to DHICA. [50] Although KA is a popular treatment for melasma, it is associated with sensitization, contact dermatitis and erythema [51]. A distinct, more stable derivative of kojic acid synthesized by Kim *et al.*, 5-[(3-aminopropyl)phosphinooxy]-2-(hydroxymethyl)-4*H*-pyran-4-one (Kojyl-APPA), showed increased skin penetration and pigment lightening efficacy in melanoma and normal human melanocytes [20,62].

### 4.7. Azelaic Acid

Azelaic acid (1,7-heptanedicarboxyilic acid, AZA) is a naturally occurring non-toxic straight chain, saturated dicarboxylic acid derived from *Pityrosporum ovale* [57,63,64]. AZA appears to selectively influence the mechanism of hyperactive and abnormal melanocytes, but minimally influences normal skin pigmentation, freckles, nevi and senile lentigenes [6,10,65]. AZA’s antiproliferative and cytotoxic effect may be mediated by the inhibition of DNA synthesis and mitochondrial oxidoreductase activity. The compound is also able to bind amino and carboxyl groups and may prevent the interaction of tyrosine in the active site of tyrosinase and thus function as a competitive inhibitor. Although not all authors are in agreement with the therapeutic efficacy of AZA, it has been reported to be effective in treatment of melasma and acne [10,65]. A 24 week multicenter, controlled, double blind clinical trial of 329 women completed by Baliña *et al.* compared the efficacy of a 20% AZA cream to a 4% HQ cream in treating melasma. The authors reported no significant difference between the results where ~65% of the patients treated with AZA were reported to achieve good to excellent results compared to ~73% of HQ treated patients [66]. 20% azelaic acid seems to be well tolerated in treated patients with no systemic side effects, but some local cutaneous irritation, a burning sensation, mild erythema, scaling and pruritus that subsided 2 to 4 weeks post treatment [65,67].

### 4.8. Gentisic Acid

The methyl ester of gentisic acid (2,5-dihydroxybenzoic acid, MG) is a natural derivative of Gentianas root with the capacity to inhibit tyrosinase. MG can act as a pro-drug that releases HQ which subsequently inhibits tyrosinase. Yet, methyl gentisate is less cytotoxic and mutagenic than HQ [10,20,50].

### 4.9. Flavonoid-like Agents

Flavonoids are natural plant polyphenols found in leaves, bark and flowers. Some 4,000 members have been identified to date, all benzo-γ-pyran derivates comprised of phenolic and pyran rings. These polyphenolic compounds are known to have anti-inflammatory, antiviral, antioxidant and anticarcinogenic properties [20,50,63]. The flavonoids may also have ROS scavenging properties and the ability to chelate metals at the active site of metalloenzymes. Flavonoids may have hypopigmenting capabilities by directly inhibiting tyrosinase activity at distal portions of the melanogenic pathway [20]. Structure-function analysis of flavonoids suggests that flavonoids with an α-keto group show potent tyrosinase inhibition due to the similarity between the dihydroxyphenyl group of DOPA and the α-keto containing flavonoids [63]. Similar analysis completed by Kubo *et al.* suggests that flavonoids have the capability to chelate copper in tyrosinase’s active site as long as the 3-hydroxygroup is free [64,68]. A comprehensive review on the properties of plant polyphenols has been published by Kim *et al.* [63] Flavonoids that will be reviewed here include aloesin, hydroxystilbene derivates and licorice extracts.

#### 4.9.1. Aloesin

Aloesin is a natural hydroxymethyl chromone compound derived from aloe vera plants [49]. It competitively inhibits the function of tyrosinase by inhibiting the hydroxylation of tyrosine to DOPA and oxidation of DOPA to dopaquinone [50]. Studies completed by Jones *et al.* on normal human melanocytes treated with aloesin, showed a dose dependent decrease in tyrosinase activity [69]. The hydrophilic nature of the compound reduces the skin penetration of aloesin. Hence, combination treatment of aloesin with arbutin has been studied to assess the synergistic effects on tyrosinase activity. The two adhere to different mechanisms of action where aloesin exhibits noncompetitive inhibition while arbutin inhibits competitively [49,64,69–71]. Testing of aloesin revealed no cytotoxicity, which makes it a good alternative to HQ [50].

#### 4.9.2. Hydroxystilbene

Some of the more efficient pigment lightening flavonoid subcategories are the hydroxystilbene compounds, derived from natural products found in oriental herbal medicines. There are more than 30 stilbene and stilbene glycosides with a structural skeleton comprised of two aromatic rings linked by an ethylene bridge [20,72]. Commonly studied hydroxystilbene products include resveratrol (3,4’,5 trihydroxystilbene), its isomer oxyresveratrol and methoxylated or glycosylated analogs piceid-glucoside, rhapontigenin and rhaponticin [20,73]. The number and position of hydroxyl substituents of hydroxystilbene compounds seem to play an important role on the inhibition of tyrosinase activity [74]. However, glycosylated hydroxystilbene compounds such as piceid, the glycoside of resveratrol at position 3, exhibit decreased tyrosinase inhibition compared to the parent compound [72]. In all cases, tyrosinase inhibition is reversible and in turn requires a high concentration of hydroxylated stilbenes within melanocytes [20]. Resveratrol, a commonly studied hydroxystilbene, is found in red wine and displays free radical scavenging, anticancer and anti-inflammatory activities [10]. Some data attribute resveratrol depigmenting affect to its ability to reduce Mitf and tyrosinase promoter activities in B16 mouse melanoma cells [18,20]. However, other contradictory results suggest that resveratrol treated normal human melanocytes (NHM) display steady-state tyrosinase RNA and, as such, regulation of tyrosinase transcription does not influence its depigmentation [75]. Additionally, further analysis of the resveratrol treated NHM displayed ER-retained immature tyrosinase, suggesting disrupted trafficking of tyrosinase in the GERL and elevated proteolytic degradation [75]. The resveratrol analog oxyresveratrol is a stronger inhibitor than resveratrol. Oxyresveratrol has been described to have a potent dose-dependent non-competitive inhibition on l-tyrosine oxidation by tyrosinase from mushroom and murine melanoma B-16, without suppression of tyrosinase synthesis or expression [74]. The hydroxystilbene products still require more analysis to properly elucidate their pigment lightening effect and mechanism(s) of action.

#### 4.9.3. Licorice Extract

Licorice extract is obtained from the root of *Glycyrrhia Glabra Linneva* and has been used in traditional Chinese medicine [6,20,48]. The main component of the hydrophobic fraction of licorice is glabridin. Glabridin has been shown to prevent UVB-induced pigmentation and to inhibit tyrosinase activity, superoxide anion production and cyclo-oxygenase activity. This suggests an influence of glabridin extract on both melanogenesis and inflammation of the skin [48,50]. Other active agents of licorice extracts include liquiritin and isoliquertin. Liquiritin is a glycoside containing flavonoid that induces skin lightening by dispersing melanin [49]. Licorice extracts also influence pigmentation by removing epidermal melanin, inhibiting the biosynthesis of melanin and inhibiting the activity of tyrosinase in a dose-dependent manner. Licorice extract has been tested in the treatment of melasma with good results and very mild irritation [50,51].

### 4.10. Antioxidants and Redox Agents

Ultraviolet radiation influences the proliferation of melanocytes and the production and secretion of paracrine and autocrine factors that stimulate melanogenesis. UV radiation can also produce reactive oxygen species (ROS) in the skin that may induce melanogenesis, DNA damage, melanocyte proliferation and/or apoptosis [76,77]. The skin contains a number of antioxidants that can be depleted by UV exposure and cause oxidative damage. The application of topical antioxidants has the capacity to prevent oxidative damage to the skin [78]. Redox agents capable of scavenging ROS generated in the skin can inhibit second messengers that may stimulate melanogenesis. Redox agents can also influence skin pigmentation by interacting with copper at the active site of tyrosinase or with o-quinones to impede the oxidative polymerization of melanin intermediates [10,79]. Here we will review vitamin C, vitamin E and later we will review the vitamin B derivative niacinamide.

#### 4.10.1. l-Ascorbic Acid and Magnesium-l-Ascorbyl-2-Phosphate

l-Ascorbic Acid (vitamin C, AsA), obtained from citrus fruits and leafy green vegetables, is a water soluble vitamin and the most plentiful antioxidant in human skin [51,80]. AsA interferes with melanin synthesis by reducing oxidized dopaquinone, interrupting DHICA oxidation and interacting with copper ions at the active site of tyrosinase. AsA acts as an ROS scavenger by donating electrons to neutralize free radicals found in the aqueous compartment of the cell [10,80]. Unfortunately, AsA is highly unstable and is rapidly oxidized and decomposed in aqueous solutions. The hydrophilic nature of AsA also limits its skin penetration, unless the stratum corneum barrier is disrupted [10,51]. The more stable ascorbate ester, magnesium-l-ascorbyl-2-phosphate (MAP) is more lipophilic and has a greater permeation through the stratum corneum [50,51]. MAP is then hydrolyzed by phosphatases in the skin to AsA and demonstrates the reducing capabilities of AsA [81]. In related skin lightening studies, Hakozaki *et al.* have used ultrasound to increase the transepidermal penetration and efficacy of the vitamin C derivative, ascorbyl glucoside and niacinamide [82].

#### 4.10.2. Alpha Tocopherol and Alpha Tocopherol Ferulate

Vitamin E (α-tocopherol, α-Toc) is a lipophilic antioxidant in the body, derived from cereal, vegetables, vegetable oil and nuts [51]. α-Toc is known to inhibit the oxidative attack of free and membrane bound unsaturated fatty acid and interferes with the lipid peroxidation of melanocyte membranes. The vitamin is also able to scavenge free radicals including superoxide anions, hydroxyl radicals and singlet molecular oxygen. It can also act as a humectant. The depigmenting effect of α-Toc can further be attributed to an increase in intracellular glutathione and the inhibition of tyrosinase [51,83–85]. α-Tocopherol ferulate (α-TF) is a related compound, in which α-tocopherol is linked by an ester bond to ferulic acid, an antioxidant that stabilizes α-Toc [10]. The presence of another antioxidant ferulic acid, causes a rapid regeneration of α-Toc and maintains a long lasting antioxidant effect. A study completed investigating the biochemical effect of α-TF in human melanoma cells suggests the whitening effect is due to tyrosinase inhibition at the post-transcriptional level, possibly by an unidentified secondary molecule [50,86].

## 5. Interruption of Melanosome Transfer

Regulation of cutaneous pigmentation is dependent on several processes beyond melanin synthesis within the melanosome. The efficiency of melanosomal transfer from melanocytes to keratinocytes, followed by melanosome processing in the recipient keratinocytes plays a critical role in skin pigmentation. Without successful transfer of melanosomes to keratinocytes, the skin can appear essentially unpigmented [87]. Treatment modalities aimed at inhibiting melanosome transfer may influence and modulate skin pigmentation.

### 5.1. Centaureidin and Methylophiopogonanone B

The first step of melanosome transfer from melanocytes to surrounding keratinocytes is successful melanocytic dendrite formation and extension towards surrounding keratinocytes. The extension of melanocytic dendrites requires the reorganization of the melanocyte cytoskeletal elements such as actin filaments and microtubules [88]. Small GTPases Rho, Rac and Cdc42 play a pivotal role in cell morphology and dendrite formation. Specifically, Rac stimulates membrane ruffling and lamellipodia formation, Rho activates dendrite retraction and Cdc42 mediates filopodia and peripheral actin microspike formation [88–90]. Ito *et al.* has shown that treatment of melanocyte and keratinocyte co-cultures with methylophiopogonanone B (5,7-dihydroxy-6,8-dimethyl-3-(4-methoxybenzyl)chroman-4-one, MOPB), an agent reported to activate Rho and induce microtubule disorganization and tubule depolymerization, appeared to reduce melanosome transfer [89,90]. The authors also showed that treatment with 1μM MOPB did not influence melanin synthesis or the expression of melanogenic enzymes. MOPB appeared to induce a reversible dendrite retraction and transfer inhibition without associated cytotoxicity (tested up to 72 hours) [89]. Centaureidin (5,7,3’-trihydroxy-3,6,4’-trimethoxyflavone), a flavonoid glucoside derived from yarrow, also reduces melanosomal transfer to keratinocytes. Centaureidin is believed to directly or indirectly activate Rho, leading to melanocyte dendrite retraction without influencing melanogenic enzyme expression or melanin synthesis [88,91]. More analysis is needed to confirm the applicability of MOPB and centaureidin as skin lightening agents.

### 5.2. Niacinamide

Niacinamide (vitamin B3, nicotinamide, 3-pyridinecarboxamide) is a biologically active form of niacin found in many root vegetables as well as in yeast [70,92]. Physiologically, niacinamide functions as a precursor to the co-factors nicotinamide adenine dinucleotide (NAD) and nicotinamide adenine dinucleotide phosphate (NADP). Along with their reduced forms NADH and NADPH, these enzymes participate in numerous enzymatic reactions and also act as antioxidants [70,92]. Niacinamide has several proposed medicinal applications in the skin including anti-inflammation, prevention of photoimmunosuppression and increased intercellular lipid synthesis [93]. Niacinamide’s role as a co-enzyme precursor may explain the multiple roles it has in skin, but this is not clearly defined [82,94]. Topical niacinamide is described to have several benefits on aging skin including but not limited to improved barrier function, improved appearance of photoaged facial skin (including texture, hyperpigmentation, redness, fine lines and wrinkles) and reduced sebum production [92,94–97]. Additionally, niacinamide is believed to influence cutaneous pigmentation by down-regulating transfer of melanosomes from the melanocytes to the keratinocytes [20,57,92]. Studies completed by Hakozaki *et al.* suggest that niacinamide has no effect on tyrosinase activity, melanin synthesis or melanocyte number in a monolayer culture system. Alternatively, the authors found that niacinamide down-regulated the number of melanosomes transferred from melanocytes to keratinocytes by 35 to 68% in a co-culture model system [92]. The actual process by which niacinamide down-regulates melanosome transfer remains to be properly established.

### 5.3. PAR-2 Inhibitors

PAR-2 belongs to a family of transmembrane G-protein coupled receptors (PAR1–PAR4) that are proteolytically activated by serine proteases. Specifically, the serine proteases (including trypsin or mast cell tryptase) cleave the extracellular amino terminal domain exposing a newly created *N*-terminus tethered ligand that undergoes a conformational change, binds and subsequently activates the receptors [98,99]. Within the epidermis, PAR-2 is expressed in keratinocytes, but not in melanocytes and is involved in the regulation of skin pigmentation through melanocyte-keratinocyte interactions [99–103]. Studies completed on keratinocyte PAR-2 indicate that it may influence melanosome incorporation and phagocytosis by keratinocytes and play a regulatory role in skin pigmentation. Therefore, modulation of PAR-2 activity augments or decreases melanosome transfer and in turn skin pigmentation [99,103–106]. PAR-2 activation can be achieved by synthetic peptides corresponding to the sequence of the *N*-terminal ligand. The peptide of mouse PAR-2 cleavage sequence SLIGRL is an equipotent activator of mouse and human PAR-2 receptor in comparison to the human tethered ligand SLIGKV [100]. Interestingly, PAR-2 expression and induction by ultraviolet irradiation is dependent on skin type, with a higher overall expression and induction in darker skin individuals [98,104,107]. Activation of PAR-2 enhances melanosome transfer, while inhibition of PAR-2 by serine protease inhibitors can result in reduced melanosomal transfer and distribution. Work published by Seiberg *et al.* and Paine *et al.* has shown that this inhibition leads to a dose-dependent lightening of skin pigmentation [103,104,106]. Work completed by Seiberg *et al.* examining the effect of serine protease inhibitor RWJ-50353 on epidermal equivalents shows an accumulation of melanosomes in melanocytes, with an increase in early stage melanosomes compared to untreated controls. They hypothesize that the keratinocytes inability to receive the presented melanosomes leads to an accumulation of melanosomes in the melanocytic dendrite and a concomitant negative feedback mechanism that slows pigment production. The authors additionally showed that Yucatan swine skin treated with RWJ-50353 for an 8 week period demonstrated a dose dependent, reversible skin lightening effect [103].

“Natural” therapies derived from soybeans have been explored for their safety and efficacy as depigmentation treatments. Two protein proteinase inhibitors have been isolated from soybean seeds Kunitz-type trypsin inhibitor (soybean trypsin inhibitor, STI) and the Bowman-Birk protease inhibitor (BBI). STI and BBI are found in the seeds of soybeans, but not in the other regions of the plant. STI inhibits trypsin proteolysis by forming a stable stoichiometric complex and BBI inhibits trypsin and chymotrypsin at separate reactive sites [106,108]. Work completed by Paine *et al.* has shown that soymilk and soybean extract reduces pigmentation in dark skinned Yucatan swine treated for an eight week period. The authors suggest that the soymilk inhibits PAR-2 activation in the skin and results in skin depigmentation. Moreover, the authors suggest that STI and BBI inhibit PAR-2 activation, causes a reduction in keratinocyte phagocytosis and a reduction in resulting skin pigmentation [106]. Soymilk also contains other constituents that may induce skin lightening such as trace amounts of free fatty acids and their acyl CoA esters that can inhibit trypsin and may participate in PAR-2 inhibition. Soybeans contain isoflavones, which are antioxidants that may reduce tyrosinase’s DOPA oxidase activity. In addition, soybeans contain phospholipids which the authors suggest may assist in the epidermal delivery of STI and BBI without the assistance of liposomes, when soy milk is used as a topical treatment [51,106].

### 5.4. Lectins and Neoglycoproteins

Cellular recognition between melanocytes and keratinocytes is an important event involved in melanosome transfer [87]. Lectins and neoglycoproteins have been explored as candidates that are involved in this phenomenon, because of their influence in cellular processes including intracellular trafficking, endocytosis and cell-cell recognition [87,93]. Interestingly, Minwalla *et al.* have demonstrated a role for melanocyte and keratinocyte membrane glycosylated residues in the process of receptor-mediated endocytosis to facilitate melanosome transfer. The authors suggest that lectins and neoglycoproteins play an inhibitory role in this process [20,87]. Specifically, plasma membrane lectins and their glycoconjugates are thought to interrupt melanocyte and keratinocyte contact and interaction, by binding their specific plasma membrane receptors, inhibiting melanosome transfer [109]. This inhibition is reversible and is shown to be enhanced by the presence of niacinamide [93].

## 6. Acceleration of Epidermal Turnover and Desquamation

The capability of a compound to accelerate the turnover of epidermal layers and/or disperse melanin pigment can result in skin lightening. Chemical agents used to exfoliate the skin, stimulates the removal of pigmented upper layer keratinocytes to lighten skin [10,110].

### 6.1. α-Hydroxyacids

α-Hydroxyacids (AHA) are weak organic acids found in fruits, plants and milk sugars [111]. For centuries, α-hydroxyacids have been one of the most commonly utilized peeling agents used to treat dry skin, acne, actinic damage and to improve skin color/texture [51]. AHAs are also reported to effectively treat pigmentary lesions such as solar lentigenes, melasma and post inflammatory hyperpigmentation (PIH). At low concentrations AHAs promotes exfoliation by decreasing corneocyte cohesion and stimulating new growth in the basal layer, while at higher concentrations AHAs promote epidermolysis and dispersed basal layer melanin. The accelerated desquamation of the stratum corneum by AHAs is complemented by a direct inhibition of tyrosinase, without influencing mRNA or protein expression [51,110,111]. Lactic acid (LA) and glycolic acid (GA) are AHAs derived from sour milk and sugarcane juice, respectively [110]. In a study investigating the histological differences between Japanese subjects treated for 6 weeks with 40% AHA, either glycolic, lactic or acetic acids, Yamamoto *et al.* reported an increase in epidermal thickness, decreased melanin deposition and up-regulated collagen levels. The authors also suggest that the AHAs induced a remodeling of the epidermis with accelerated desquamation [112].

### 6.2. Salicylic Acid

Salicylic Acid (SA) is a β-hydroxyacid found in willow bark and sweet birch. It is also a phytohormone, a plant product that acts similar to a hormone and regulates cell growth and differentiation. SA functions as a desquamating agent that penetrates and dissolves the intercellular matrix of the stratum corneum [50,111].

### 6.3. Linoleic Acid

Unsaturated fatty acids including oleic acid (C18:1), linoleic acid (C18:2) or α-linolenic acid (C18:3) suppresses melanogenesis and tyrosinase activity, while saturated fatty acids such as palmitic acid (C16:0) or stearic acid (C18:0) increases it [113]. Linoleic acid reduces the activity of tyrosinase in melanocytes, while mRNA levels remain unchanged [51]. No evidence of change in TYRP-1 and TYRP-2 protein levels suggest that fatty acids selectively target tyrosinase. This may influence the enzyme’s degradation via a physiologic proteasome-dependent mechanism, altering the tyrosinase protein content in hyperactive melanocytes [10,114]. Linoleic acid also influences skin pigmentation by stimulating epidermal turnover and increased desquamation of melanin pigment from the epidermis [51]. Studies completed to assess the skin lightening capabilities of unsaturated fatty acids, linoleic acid or α-linoleic acid, on UV induced hyperpigmentation of brown guinea pig skin, showed an efficient lightening effect [115]. It is thought that the unsaturated bonds of these molecules can be easily peroxidized, which in combination with an increase in epidermal turnover, correlate with an inhibitory effect on melanogenesis *in vivo* [51,115].

### 6.4. Retinoids

Retinoids are a common treatment option used to ameliorate acne, photodamage and PIH. The mechanism of action likely involves the inhibition of tyrosinase, the dispersion of keratinocyte pigmented granules, reduction in pigment transfer and a reduction in corneocyte cohesion with an associated acceleration of epidermal turnover [20,51,116]. Tretinoin (*all*-trans retinoic acid) is a derivative of vitamin A that is thought to have an inhibitory effect on tyrosinase transcription [117]. Tretinoin is reported to be effective in treating melasma, with some associated side effects including erythema, peeling at the site of application and PIH [20]. Tretinoin is also used in combination in topical creams, such as a formulation proposed by Kligman and Willis containing 5% HQ, 0.1% tretinoin and 0.1% dexamethasone. Tretinoin in this formulation acts as a stimulant of epidermal turnover and pigment reduction via epidermopoieses, an antioxidant to reduce the oxidation of HQ and a mild irritant to enhance the epidermal penetration of HQ [110].

## 7. Conclusions

Great advances have been made in understanding the cellular and biochemical mechanisms in pigment biology and the processes underlying skin pigmentation. This has led to the development of various skin lightening agents to reduce skin hyperpigmentation. While several agents target the rate limiting enzyme of melanogenesis tyrosinase, there has been an increased interest in alternative hypopigmenting mechanisms. Yet, as addressed by Lei *et al.*, there is a need for a standardized and streamlined protocol to screen melanogenic regulatory compounds, to simplify the difficult task of product comparison [118]. Also, as the number of putative depigmenting agents grows there is an increased need for studies to clarify product efficacy, cytotoxicity, topical skin penetration, stability and safety. To add to the complexity, it may be more advantageous to test compounds together to address the synergistic effects on skin lightening, particularly when the active components influence distinct steps of melanogenesis [10]. While it is clear that great progress has been made in the study of skin lightening, it is even more apparent that there is a great deal of work still left to be done.
